# The Global Burden of Trachoma: A Review

**DOI:** 10.1371/journal.pntd.0000460

**Published:** 2009-10-27

**Authors:** Matthew J. Burton, David C. W. Mabey

**Affiliations:** Department of Infectious and Tropical Diseases, London School of Hygiene and Tropical Medicine, London, United Kingdom; London School of Hygiene & Tropical Medicine, United Kingdom

## Abstract

Trachoma is the commonest infectious cause of blindness worldwide. Recurrent infection of the ocular surface by *Chlamydia trachomatis*, the causative agent, leads to inturning of the eyelashes (trichiasis) and blinding corneal opacification. Trachoma is endemic in more than 50 countries. It is currently estimated that there are about 1.3 million people blind from the disease and a further 8.2 million have trichiasis. Several estimates for the burden of disease from trachoma have been made, giving quite variable results. The variation is partly because different prevalence data have been used and partly because different sequelae have been included. The most recent estimate from the WHO placed it at around 1.3 million Disability-Adjusted Life Years (DALYs). A key issue in producing a reliable estimate of the global burden of trachoma is the limited amount of reliable survey data from endemic regions.

## Author Summary

This review examines the various attempts to estimate the burden of disease from trachoma, the commonest infectious cause of blindness worldwide. Reports vary considerably because of differences in methodology and changing estimates of the number of people affected. Currently about 1.3 million are blind from trachoma and it causes about 1.3 million Disability-Adjusted Life Years (DALYs). The limited amount of survey data available from endemic regions remains a problem in generating accurate estimates. The effect of the disease may be underestimated as some of the disabling sequelae are not included in the calculation.

## Introduction

Trachoma is the leading infectious cause of blindness worldwide. Overall it is the eighth commonest blinding disease [1]. Trachoma is caused by the obligate intracellular bacterium *C. trachomatis*. Recurrent episodes of conjunctival infection and the associated chronic inflammation it causes initiate a scarring process that ultimately leads to irreversible blindness. There is a worldwide effort underway to try to control blinding trachoma; this is lead by the World Health Organization (WHO) with the Global Alliance for the Elimination of Blinding Trachoma (GET2020).

It is estimated that approximately 1.3 million people are blind from this disease and probably a further 1.8 million have low vision [2]. Trachoma is endemic in more than 50 countries, predominantly in sub-Saharan Africa, the Middle East, and Asia [3]. The burden of trachoma on affected individuals and communities can be huge both in terms of the disability it causes and the economic costs that result. In this paper we review the available data on the prevalence of the disease and estimates of its burden.

## Pathogenesis and Clinical Features

Endemic trachoma is caused by the four ocular serovars of *C. trachomatis* (A, B, Ba, and C). Although the genital serovars (D to K) of *C. trachomatis* can infect the conjunctiva causing either ophthalmia neonatorum in infants or inclusion conjunctivitis in adults these are usually isolated episodes for the individual, which do not lead to blinding sequelae. For endemic trachoma the average age of acquisition of the first episode of *C. trachomatis* infection is probably related to the prevailing level of infection in the community. In hyperendemic settings infection may be acquired in early infancy, whereas in meso- and hypo-endemic regions it is probably on average later. Infection is probably usually acquired through living in close physical proximity to an infected person, with the family as the principle unit for transmission [4],[5].

**Figure 1:**
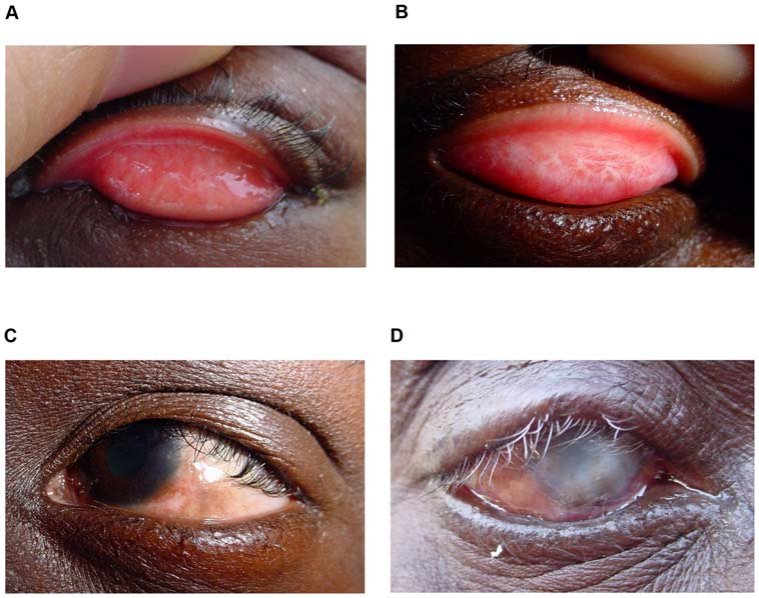
Clinical features of trachoma. (A) Active trachoma in a child, characterised by a mixed papillary (TI) and follicular response (TF). (B) Tarsal conjunctival scarring (TS). (C) Entropion and trichiasis (TT). (D) Blinding CO with entropion and trichiasis (TT).

Conjunctival infection with *C. trachomatis* is largely confined to the epithelium, with little evidence of deeper spread. The infection triggers an immune response characterised by a marked inflammatory cell infiltrate and the release of pro-inflammatory cytokines in the conjunctiva [6],[7]. Clinically it causes papillary and/or follicular inflammation of the tarsal conjunctiva, referred to as active trachoma (Figure 1A). The WHO Simplified Trachoma Grading System (Table 1), which is used by trachoma control programmes, subdivides active trachoma into two often coexisting clinical phenotypes: Trachoma Inflammation Follicular (TF) and Trachoma Inflammation Intense (TI) [8]. Eventually the infection resolves and the clinically visible inflammation gradually subsides. Animal models for *C. trachomatis* infection and limited data from humans indicate that the resolution of infection is probably through an interferon-γ (IFN-γ)–dependent cell-mediated immune response [9],[10]. Studies from trachoma endemic communities have found that the prevalence and duration of conjunctival chlamydial infection decline with increasing age, suggesting that there is a maturation of the immune response as individuals are repeatedly exposed to infection [11],[12]. However, in the early vaccine trials using whole *C. trachomatis* organisms the acquired immunity appeared to be strain specific and relatively short-lived [13]–[15]. As a result of the relatively ineffective immune response, repeated infection of the individual by *Chlamydia trachomatis* is common within an endemic environment. This leads to a recurrent chronic inflammation, which is associated with the development of scar tissue within the conjunctiva over many years (Figure 1B) [16],[17]. As the scar tissue accumulates it also contracts, causing the eyelids to roll inwards towards the eye (entropion) and the eyelashes to scratch the ocular surface (trichiasis, Figure 1C). The degree to which conjunctival scarring develops probably depends on a complex interaction between the pressure of infection (load and frequency) and host specific immunogenetic factors [18]–[20]. It is possible that a failure of the immune response to adequately control the chlamydia leads to prolonged infection episodes, which provokes more severe inflammation, tissue damage (through the release of proteases), and aberrant repair [7]. The most serious disease sequela from trachoma is blinding corneal opacification (CO, Figure 1D). The main aetiological risk factor for corneal damage is the presence of trichiasis, however, a number of other factors probably contribute such as bacterial infection and chronic conjunctival inflammation [21].

**Table 1:** The WHO simplified system for the assessment of trachoma [8].

Grade	Abbreviation	Description
**Trachomatous inflammation – follicular**	TF	The presence of five or more follicles (>0.5 mm) in the upper tarsal conjunctiva
**Trachomatous inflammation – intense**	TI	Pronounced inflammatory thickening of the tarsal conjunctiva that obscures more than half of the deep normal vessels
**Trachomatous scarring**	TS	The presence of scarring in the tarsal conjunctiva
**Trachomatous trichiasis**	TT	At least one lash rubs on the eyeball
**Corneal opacity**	CO	Easily visible corneal opacity over the pupil

## Epidemiology

**Figure 2:**
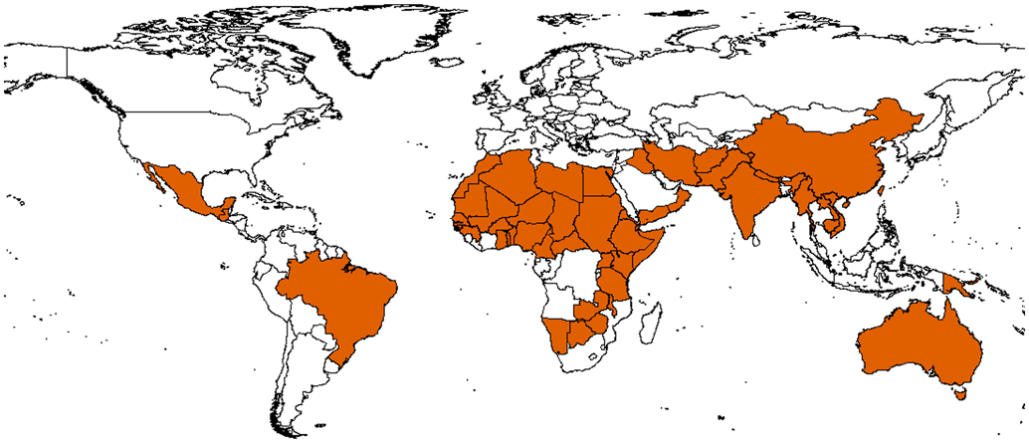
Map of trachoma endemic countries in 2009. Reproduced with permission from Silvio P. Mariotti, WHO/NMH/PBD.

Today, blinding trachoma is believed to be endemic in 56 countries (Figure 2) [22]. The countries with the highest prevalence of disease are in sub-Saharan Africa, particularly in the Sahel belt and East Africa. In addition, there are countries in the Middle East, the Indian sub-Continent, and Southeast Asia where trachoma is endemic, although the distribution is patchier. One hundred years ago trachoma was widespread in Europe and North America, but faded away during the first half of the 20th century as living conditions improved [23].

The clinical manifestations of trachoma change with age. Active trachoma is predominantly seen in young children, becoming less frequent and shorter in duration with increasing age [11],[12]. Conjunctival scarring accumulates with age, usually becoming evident in the second or third decade of life [24],[25]. Entropion, trichiasis, and CO develop later. The onset of the blinding complications of trachoma can occur in children living in regions where the pressure of infection is high [26]. Epidemiological surveys have generally found trichiasis and CO to be more common in women than men [25],[27]. This difference has been attributed to the greater life time exposure of women to *C. trachomatis* infection, through closer contact with children, the main reservoir of infection.

The transmission of *C. trachomatis* from infected to noninfected individuals is necessary to sustain trachoma in endemic communities. Several routes of transmission are probably involved including direct spread (close contact), fomites, and eye-seeking flies. In common with other Neglected Tropical Diseases (NTDs), trachoma is generally a disease of resource-poor rural communities. Risk factors for trachoma are generally things that favour the transmission of *C. trachomatis* from one person to another [28]. The presence of secretions around the eyes has consistently been associated with active trachoma, attracting flies, and providing a vehicle for transmission. Similarly, water scarcity probably promotes transmission, because less water is available to use for face washing. Limited access to latrines increases faecal contamination of the environment, providing breeding material for the fly *Musca sorbens*, which is implicated in trachoma transmission [29]. Crowded living conditions, for example with several young children sleeping in the same bed, probably promotes transmission. For many trachoma endemic countries the socioeconomic developments that might promote the disappearance of the disease are likely to be very slow in arriving, which in the light of demographic trends and in the absence of effective control programmes was predicted to lead to an increase in the total numbers blind from trachoma [30].

## Global Prevalence of Trachoma

Several estimates of the number of people affected by trachoma worldwide have been made by the WHO and others (Table 2) [2], [3], [31]–[35]. These estimates have generally been produced with models that have relied on the results of a limited number of surveys conducted in a few endemic countries. The results have been extrapolated across these countries and to other countries in the same region. However, there are problems. The list of countries included has sometimes been incomplete or contained countries without endemic trachoma. The survey data used is sparse, often old, and some of it is of questionable reliability. In the WHO estimate from the 1990s there was probably a significant overestimate of the number blind from trachoma (6 million), because part of the estimate was based on questionnaires reporting numbers of people who might become blind without treatment [31],[33]. Moreover, there are big gaps. In particular, there are limited data from both India and China, where pockets of the disease are thought to exist; even with a low prevalence the contribution from these two most populous countries could make a profound difference in the global burden of trachoma. More recent estimates have been more stringent, using only surveys with a national sampling frame and the updated WHO list of endemic countries [33].

**Table 2:** Estimates of the number of individuals affected by trachoma worldwide since 1981.

Year Estimated	Active Trachoma	Trichiasis	Blind	Low Vision
**1981 ** [**32**]	500	—	2	—
**1990 ** [**34**]	—	—	2.9	3.8
**1990 ** [**31**]	—	—	5.9	—
**1990 ** [**33**]	—	—	2.8	3.9
**1991 ** [**35**]	146	10	—	—
**2000 ** [**33**]	—	—	3.8	5.3
**2002 ** [**2**]	—	—	1.3	—
**2003 ** [**3**]	84	7.6	—	—
**2008 ** [**36**]	40	8.2	—	—

Numbers are millions of people.

However, despite the limitations and potential unreliability of the available data, there does appear to be an encouraging downward trend in the numbers (Table 2). The most recent estimate, released in 2008, suggests that there are currently about 40 million people with active trachoma and 8.2 million with trichiasis [36]. The significant downward revision in this estimate was attributed in large part to significant overestimates in the number of cases in China and India in the 2004 figures. The highest prevalence of trachoma is reported from Ethiopia and Sudan, where active trachoma is often found in more than 50% of children under 10 y, and trichiasis is found in up to 19% of adults [37],[38].

## Global Burden of Trachoma

The disabling sequelae of trachoma are visual impairment and trichiasis. These have a major advantage over the disease sequelae reported for many other NTDs as they can be readily measured or observed clinically and do not require special investigations. Visual impairment is subdivided into Blindness and Low Vision (Definitions: Box 1). Visual impairment can have a profound impact on many aspects of the life of an individual (mobility, psychological, social, financial, mortality), their family, and the wider community. Trichiasis can also be disabling in the absence of visual impairment by causing pain and photophobia. A study from Tanzania found that women who had trichiasis without visual impairment suffered a degree of disability that was comparable to that caused by visual impairment (without trichiasis) from causes other than trachoma [39]. Moreover, when both trichiasis and visual impairment are present the degree of limitation rises to roughly double that of either of these two elements alone. However, despite the evidence of disability caused by trichiasis, only one estimate of the burden of disease from trachoma has included it [40].

**Box 1. Definitions of Blindness, Low Vision and Visual Impairment (International Statistical Classification of Diseases [ICD-10]) [57]**

- Blindness: visual acuity of less than 3/60 (20/400), or a visual field loss to less than 10° from fixation in the better eye with best possible correction (ICD-10 visual impairment categories 3, 4, and 5).
- Low Vision: visual acuity of less than 6/18 (20/60) but equal to or better than 3/60 in the better eye with best possible correction (ICD-10 visual impairment categories 1 and 2).
- Visual Impairment: combines both Blindness and Low Vision (ICD-10 visual impairment categories 1–5).

The first attempt to calculate the burden of trachoma was in the Global Burden of Disease study (GBD) [41]. The GBD study developed a new measure of the burden of disease: Disability-Adjusted Life Years (DALYs). This measures the gap, in terms of healthy life lost, between an “ideal” healthy population and the reality caused by a specific disease in terms of premature mortality and disability in a particular society. DALYs and the GBD study have been discussed in detail elsewhere [42]. In essence there are two major components in the calculation of DALYs: a measure of premature mortality, years of life lost (YLL); and a measure of years of healthy life lost through disability caused by the disease, years of life lived with a disability (YLD). Different disabilities are weighted so that the more severe the disability the greater the number of YLDs that are lost. In order to make the calculation it is necessary to have estimates of the number of people dying from the disease or living with the disability for a given year.

The GBD study estimated the burden of trachoma to be about 1.0 million DALYs annually, the vast majority of which was due to YLD (Table 3). This estimate used WHO figures for the number of people affected by trachoma at that time, which were probably somewhat less accurate than subsequent estimates, as discussed above. Moreover, the list of endemic countries used in the process probably left out some now known to be endemic and included others where there is not thought to be a problem. Subsequently, as part of the ongoing updates to the GBD project there have been further estimations of the burden of trachoma by the WHO included in the *World Health Report* using updated estimates for the global prevalence of the disease (Table 3).

**Table 3:** Estimates of the Global Burden of Trachoma.

Year	Source	Blindness Cases	Low Vision Cases	Blindness Weight	Low Vision Weight	Burden	
						DALY or HALY	*n*
**1990**	**WDR ** [**41**]	—	—	—	—	DALY	1,024,000
**1990**	**Evans ** [**43**]	2.9	3.8	0.58	0.59	Lifetime HALY	79,509,000
**2000**	**Frick ** [**33**]	3.8	5.3	0.600	0.245	DALY	3,565,725
**2000**	**Frick ** [**33**]	3.8	5.3	0.600	0.245	Lifetime DALY	38,887,187
**2002**	**WHO/GBD ** [**58**]	2.9	3.5	0.600	0.278	DALY	2,329,000
**2004**	**WHO/GBD ** [**59**]	—	—	0.600	0.278	DALY	1,334,000

DALYs and HALYs are not directly comparable, as discussed in the text.

There have been two other calculations of the burden of disease from Trachoma, in addition to the GBD programme. The first of these, by Evans and Ranson, was published in 1995 and was an estimate of the Handicap-Adjusted Life Years (HALYs) for the year 1990 (Table 3) [43]. This measure is similar in concept to the DALY, consisting of a composite of the number of years lost through an early death and the number of years lived in a handicapped state. Instead of using disability weights the authors developed a new measure, the handicapped weight. The handicapped weights were estimated from self-assessment questionnaires of people with trachomatous visual impairment. In addition, to produce this estimate the investigators performed a fresh calculation for the number of people blind or with low vision from trachoma worldwide [34]. The value of handicapped weight for blindness they used was similar to that for DALY calculations (0.58). However, the value for the low vision handicapped weight (0.59) was higher than the disability weight for low vision used in other estimates (0.27). They also performed a sensitivity analysis that found a high degree of uncertainty around the mean estimate of 79.5 million lifetime HALYs, with the 95% confidence interval (CI) ranging from 15 to 500 million. This reflects the high degree of uncertainty both in the estimates of prevalence and other parameters.

The second estimate was made by Frick and colleagues for the year 2000 (Table 3) [33]. In this calculation the investigators excluded YLL, because they did not consider its estimate to be sufficiently reliable. They used a relatively low disability weight for low vision (0.245). They re-estimated the number of people with blindness from trachoma worldwide on the basis of a reassessment of survey data collected since 1980. On the basis of previous published data they calculated that for each person blinded by trachoma there were a further 1.4 with low vision, and extrapolated the number of people with low vision on this basis. Their estimate of the annual DALYs was 3.6 million, with 72% of these DALYs occurring within sub-Saharan Africa and 80% of lifetime DALYs occurring in women. This figure was significantly higher than the GBD estimate for the same year (2.2 million).

Estimates of the burden of trachoma suffer from several weaknesses. The first is the limited supply of reliable data on the prevalence of disease sequelae in endemic populations. There are relatively few robust population-based surveys that can be used to estimate the number of affected people. The more recent estimates of burden have benefited from the work done by the WHO/GET2020 in defining the list of countries that currently have endemic trachoma. Secondly, there is uncertainty over whether to include trichiasis as a disabling disease sequela, independent of visual impairment. One report has suggested that the additional disability caused by trichiasis, independent of visual impairment, may add as much as 50% to the burden of disease caused by trachoma [40]. Finally, it remains unclear, due to an absence of data, what degree of premature mortality trachoma-related blindness may cause. There are two studies from rural communities in sub-Saharan Africa that have examined the question of excess mortality due to visual impairment [44],[45]. Both of these found an increase in mortality amongst blind people compared to sighted controls. As the potential for confounding in assessing mortality related to visual impairment is high, it is necessary to conduct carefully designed studies to investigate this question specifically for trachoma.

## Economic Cost of Trachoma

Two estimates of the economic cost of trachoma have been made by Frick and colleagues [33],[40]; these are framed in terms of lost productivity. The economic cost of one disabled person was calculated by multiplying the value of the disability weights by the individual economic productivity value for each country considered. In the first estimate only the burden caused by visual impairment was considered and the productivity lost was estimated at US░ 2.9 billion (1995) [33]. In the second estimate it was found that the economic loss was higher at US░ 5.3 billion (2003) [40]. This second estimate used the adjusted dollar value for 2003, it considered the productivity lost from blindness to be 100% (instead of 60%) and it added a 10% cost for each blind person for a carer. The investigators also examined the effect of including trichiasis and found that the lost productivity rises significantly to US░ 8 billion.

There are significant challenges in producing meaningful estimates of the economic cost of trachoma. Estimates are made of the cost to the world economy as a whole, rather than the relative cost to the affected individual. They are greatly influenced by the per capita GDP of each country involved. A particular difficulty is determining what average individual productivity value to use. In the estimates described above the authors used (where available) the “average agricultural value added per worker.” However, this was not available for each country. It is also assumed that the affected individual would always be in full-time paid employment in the absence of their disability and no adjustment of potential earnings is made for age or sex. It is also difficult to include a measure for the productivity lost outside the formal work place or the indirect cost to the carer of a visually impaired person. Finally there are issues around the choice of which disabling sequelae to include and the disability weights these should be given, discussed above.

## Trachoma Control and Its Cost-effectiveness

There have been organised trachoma control programmes in many endemic countries for decades, which have met with variable success. In 1998 the World Health Assembly resolved to eliminate blinding trachoma by the year 2020 [46]. To this end the GET2020 was formed, including representatives from the WHO, national blindness control programmes from endemic countries, nongovernmental organisations working in the field, industry, and academic institutions. The GET2020 alliance adopted the SAFE Strategy as its favoured approach to controlling trachoma [47]. The four components of SAFE are surgery for trichiasis, antibiotics for infection, facial cleanliness, and environmental improvements to reduce transmission.

There is a growing body of published evidence supporting the clinical effectiveness of each component of the SAFE strategy [48]. In the case of trichiasis surgery there are currently nine published randomised controlled trials investigating various aspects of management including the optimal type of operation, level of surgeon, where surgery should be done, and whether peri-operative antibiotic improves the outcome [49]. However, there are major problems both with the outcome of surgery, with high trichiasis recurrence rates reported under operational conditions, and the effective delivery of the service on the ground in many endemic countries [21],[50].

Over the last 60 y there have been many studies testing different antibiotics for active trachoma. The WHO currently recommends the use of either oral azithromycin (single dose) or topical tetracycline (twice daily for 6 wk). Both of these antibiotics have been demonstrated to be effective in clinical trials at reducing the prevalence of both active disease and *C. trachomatis* infection [51]. The clinical signs of active trachoma have a relatively low sensitivity for the identification of *C. trachomatis* infection. In addition, *C. trachomatis* has the potential to rapidly reemerge in communities where some infected cases are left untreated. Therefore, the current recommendation is for mass drug administration (MDA) of entire endemic communities to be conducted annually for several years, until the prevalence of follicular trachoma (TF) in children ages 1–9 y drops below 5% [3]. It is likely that, with such a low threshold for treatment, very large numbers of uninfected people will be treated with antibiotic in order to catch all those harbouring infection. The evidence base supporting the effectiveness of face washing and environmental interventions in reducing trachoma is more limited [48]. However, the historical epidemiology of trachoma strongly supports the view that general improvements in hygiene can have a profound long-term effect on this disease.

Several investigators have produced estimates of the cost-effectiveness of trachoma control programmes or individual components of the SAFE strategy. The only study evaluating the long-term cost-effectiveness of an entire national trachoma control programme was made by Evans and colleagues for Myanmar (Burma) over a 30-y period (1964–1993) [52]. This programme predated the introduction the SAFE strategy; however, it contained several elements of today's trachoma control programmes: surgery, mass antibiotic distribution, and community education. During this period there was a marked decline in the disease in Myanmar. The overall cost-effectiveness of the programme was estimated at US░54 per case of visual impairment prevented. Two factors may have lead to an overestimate of the cost-effectiveness: (1) it was assumed that all the visual impairment from trachoma prevented was due to the activities of the programme, rather than to any underlying secular trend due to socioeconomic changes, (2) only the direct costs to the national programme were included.

Three analyses for the cost-effectiveness of trichiasis surgery have been produced, which consistently found it to be very cost effective. In the analysis of the Myanmar programme the average cost (over the 30-y period) per case of visual impairment prevented was estimated to be US░193, although in the last 10 y of the programme there was a marked rise in the cost-effectiveness of trichiasis surgery to US░41 per case of visual impairment prevented [52]. The cost per HALY saved was on average US░10, dropping to US░3 for the final 10 y. In The Gambia the cost of surgery was estimated to be US░6.13 per operation (1998), whilst the estimated life-time loss of economic productivity was US░89 [53]. In a separate analysis the cost-effectiveness of surgery was estimated for seven trachoma endemic world regions [54]. The cost of trichiasis surgery was estimated to be about International Dollars (I░) 19 per case in Africa. It was estimated that if surgery was carried out on 80% of the current cases of trichiasis this would save 11 million DALYs globally each year. Surgery was found to be very cost effective with estimates ranging from I░13 to I░78 per DALY, depending on the region.

The cost-effectiveness of antibiotic treatment has also been considered in a number of analyses. In the evaluation of the Myanmar programme it was found that the cost of nonsurgical interventions (mostly antibiotic treatment) was US░47 per case of visual impairment prevented [52], which gave a cost-effectiveness of US░3 per HALY averted. This seems to be a remarkably low cost and there may have been some major methodological biases that attributed the vast bulk of the DALYs to the nonsurgical as opposed to the surgical components of the programme. In contrast, the more recent projection of the cost-effectiveness of trachoma control in seven world regions found antibiotic treatment to be relatively cost ineffective [54]. For example in Africa the cost of mass antibiotic distribution of azithromycin to children aged 1–10 y was I░9,012 per DALY saved if the azithromycin had to be purchased at the standard cost price. Azithromycin is currently donated by the manufacturer, Pfizer Inc., to trachoma control programmes in 15 endemic countries. Even if the drug is donated, the authors concluded that costs remain high at I░3,922 per DALY. However, this study made a number of questionable assumptions that cast doubt on these figure. For example, the authors assumed that mass treatment would need to be given annually for 10 y, which is probably much longer than would be needed if high coverage levels are achieved. The effectiveness of mass azithromycin treatment is variable, although several studies have suggested that *C. trachomatis* infection can be well controlled with one or two rounds of mass treatment [55],[56]. The authors further assumed that the reduction in trichiasis prevalence due to mass treatment would not be seen for 45 y, and that the proportionate reduction in trichiasis and blindness would be the same as the reduction in the prevalence of active trachoma seen after 10 y. It is not known how effective controlling *C. trachomatis* infection will be on the development of the scarring sequelae in people who have previously been repeatedly infected, however it is anticipated that as the prevalence of infection drops so the drive to disease progression lessens. There are major logistical and financial obstacles, even with donated azithromycin to repeatedly conducting mass drug administration, especially in remote rural settings. In response to this there has been a move to try, where appropriate, to combine mass drug administration with azithromycin for trachoma with treatments for other NTDs.

## Conclusions and Future Directions

Several attempts have been made to estimate both the burden and cost of trachoma. It remains a significant problem with a high burden of disability. Encouragingly the reported numbers of people affected by trachoma appears to be steadily declining. However, current burden estimates are limited in reliability because of the paucity of survey data available on which to base estimates of the total number of cases. There is also variability over whether to include trichiasis, in the absence of visual impairment. In order to develop more robust estimates of the burden of trachoma there needs to be a coordinated effort to conduct population-based surveys with a national sampling frame in representative countries from endemic regions. Clarification of the situation within India and China is particularly important, given the size of their populations. A consensus also needs to be reached on whether trichiasis should be included in the calculation of DALYs and what weight this should be given. There is limited evidence of premature mortality due to blindness in general. Further studies on this specifically in relation to trachoma would be of value.
